# Cutting Force during Surface Layer Milling of Selected Aluminium Alloys

**DOI:** 10.3390/ma13245725

**Published:** 2020-12-15

**Authors:** Magdalena Zawada-Michałowska, Jerzy Józwik, Stanisław Legutko, Dariusz Mika, Paweł Pieśko, Jarosław Pytka

**Affiliations:** 1 Faculty of Mechanical Engineering, Lublin University of Technology, 20-618 Lublin, Poland; j.jozwik@pollub.pl (J.J.); p.piesko@pollub.pl (P.P.); j.pytka@pollub.pl (J.P.); 2 Faculty of Mechanical Engineering, Poznan University of Technology, 60-965 Poznań, Poland; stanislaw.legutko@put.poznan.pl; 3 Institute of Technical Sciences and Aviation, The State School of Higher Education in Chelm, 22-100 Chełm, Poland; dmika@pwsz.chelm.pl

**Keywords:** aluminium alloys, cutting force, milling, surface layer

## Abstract

This paper presents the analysis of cutting force during surface layer milling of selected aluminium alloys, which are widely used in the aviation industry. The cutting force is one of the most important parameters determining the machinability of the material and also provides important information about the course of the cutting. The study analysed the influence of the technological parameters, i.e., cutting speed *v_c_* and depth of cut *a_p_* as well as the relation between cutting tool feed direction and rolling direction on the value of cutting force during milling of selected aluminium alloys, i.e., EN AW-2017A T451 and EN AW-2024 T351. The material anisotropy is a very important issue, since the engineering industry faces enormous problems related to the cutting of the tested materials that are usually supplied in the form of rolled plates. The surface layer was cut due to the fact that it accumulates the greatest residual stresses. The measurement process of cutting force was performed by using 9257B Kistler piezoelectric dynamometer. As part of the analysis of the results, the measurement uncertainty was also estimated, which was determined on the basis of two components obtained by using the A and B methods, respectively.

## 1. Introduction

The requirements related to, among others, with weight reduction while maintaining both high strength and stiffness are imposed on thin-walled aircraft parts. When manufacturing such parts, major difficulties arise in retaining appropriate dimensional and shape accuracy, since undesirable deformations appear after the completion of cutting and removal of the clamping force [1,2]. For this reason, measuring the cutting force, which is one of the factors connected to the aforementioned problem is an important aspect [3]. It should also be noted that due to the need to obtain high efficiency, High Speed Cutting is a widely used technique which is characterised by lower depth of cut *a_p_* and higher cutting speed *v_c_* compared to conventional machining [4].

Taking into account the fact that during cutting, the residual stresses are generated primarily as a result of the cutting force, the analysis of the obtained cutting force values with different technological parameters may be of vital importance for maintaining the required dimensional and shape accuracy [5]. Therefore, it is assumed that during machining, the main model of residual stress formation is the mechanical model. Apart from the cutting force mentioned, the residual stresses result from a number other factors, e.g., clamping force, temperature, etc. [6].

The cutting force is a parameter that determines the machinability of the material, and also provides important information related to the course of the cutting process [7]. On its basis it is possible to decide to change the technological parameters [8] as well as to state that the cutting blades have become worn [9]. Liang et al. [9] also noted that the cutting force provides information about the physical mechanics and dynamics of cutting processes. That is why measurement of the cutting force, especially in real time (“online”), is so significant [10].

It should be emphasised that the cutting force depends on many factors, including, for example, technological parameters [11,12], the properties of the workpiece material, tool material and geometry as well as the cooling method used [13].

Prediction of cutting force is a key issue, especially in terms of increase in production efficiency by maximising the volume of material removed. The dynamic development of numerical methods implies a search for theoretical ways of the cutting force determination. However, due to the complexity of the phenomena accompanying the cutting process, it is difficult to apply it in practice. A mechanistic model of the prediction of milling forces for serrated milling tools was presented in [14]. This type of cutting tool is increasingly used in 5-axis high-performance machining of complex parts. The proposed model applies to both static and dynamic conditions and it allows to take into account, for example, tool parameters and technological parameters. Tsai et al. [15] showed a new geometric force prediction model for ball-end cutter. It described, among others, tool geometry (rake angle), cutting speed and chip flow angle. Urbikain Pelayo [16] presented modelling of static and dynamic milling forces for circle-segment mills. Tool geometry, modal parameters and cutting condition are taken into account. 

Currently, cutting force measurements are carried out mainly by two methods: indirect and direct. In the indirect method, the power or current of the spindle motor or the drive motor are measured and the cutting force is calculated on their basis. The direct measurements are performed when the measuring instrument is placed on the table or on the machine tool spindle and for this purpose a dynamometer is used, which is characterised by high repeatability and stability. Its principle of operation is to convert the cutting force into deformation and detect this deformation with flexible sensing elements. Currently, the most widely used are dynamometers, which in the case of instruments mounted on tables, are characterised by the fact that the flexible element has the shape of, among others: an octagonal ring, an oval octagonal ring or a ring. However, in dynamometers mounted on the machine tool spindle, the elastic part is a cylindrical beam, a Γ beam-type or an E-type diaphragm [10,17,18]. As for the type of transducer, there are, inter alia, capacitive, strain gauge, piezoelectric and fibre Bragg grating (FBG) [19,20,21]. Due to the dynamics of the cutting processes and the subsequent signal analysis, piezoelectric dynamometers are currently used to measure the cutting forces [22]. 

Aluminium alloys have different machinability compared to other materials. It results mainly from their properties, such as: high coefficient of linear expansion, low Young’s modulus and high thermal conductivity (in relation to steel). For aluminium alloys, the value of the cutting force is assumed to be about 30% of the value occurring in the machining of steel, but its precise determination requires experimental tests [23]. 

Rolled plates are widely used in manufacturing of thin-walled elements from wrought aluminium alloys. They are characterised by the phenomenon of anisotropy, i.e., having different mechanical properties depending on the rolling direction. In addition, the aforementioned anisotropy may cause deformations of parts with thin walls. It is so important to monitor cutting force in precision machining of these elements [24]. Zawada-Michałowska et al. [25] presented the removal of the textured surface layer as one of the pre-machining methods. This operation has a positive effect on minimising deformations of thin-walled elements. 

The aim of the paper is to assess the changes in the cutting force as a function of cutting speed *v_c_*, in particular to determine the minimum cutting force values that could occur during pre-machining consisting in removing the textured surface layer formed after the rolling process of plates made of wrought aluminium alloys. This is a very important issue in terms of the aforementioned residual stresses.

## 2. Materials and Methods 

**Figure 1:**
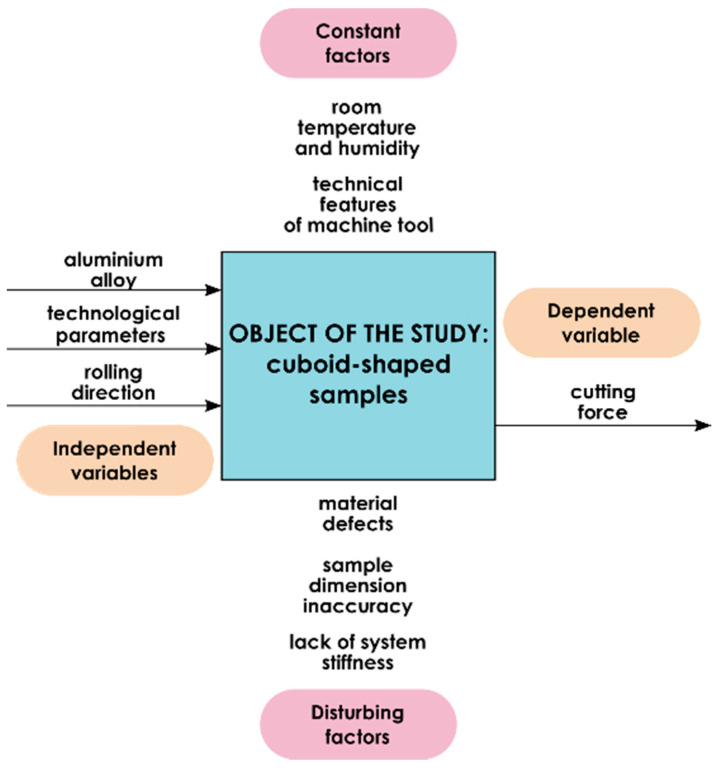
Research plan.

Figure 1 presents the research plan. The cuboid-shaped samples of the following dimensions: 20 mm × 20 mm × 64 mm were defined as the research object. The independent variables were technological parameters, rolling direction and aluminium alloy, while the dependent variable is cutting force. The constant factors include the technical features of the machine tool as well as temperature and humidity of the laboratory room. The disturbing factors were material defects, dimensional inaccuracy of samples and lack of system stiffness. 

The tests were performed with the Avia VMC 800HS vertical machining centre (Warsaw, Poland). The machined materials were EN AW-2017A T451 and EN AW-2024 T351 wrought aluminium alloys. They are widely used in the aviation industry. EN AW-2017A T451 has high strength properties—high tensile strength and fatigue strength. It is not very resistant to corrosion and less susceptible to welding. EN AW-2024 T351 has very high strength and high fatigue strength, but it has low corrosion resistance and low weldability. The chemical composition and selected properties of the EN AW-2017A T451 alloy are presented in Table 1, while for the EN AW-2024 T351 alloy in Table 2.

The values of the technological parameters used are presented in Table 3. 

Another analysed variable was rolling direction, i.e., the relation between the cutting tool feed direction and the rolling direction:

- perpendicular direction (milling direction was perpendicular to rolling direction),
- parallel direction (milling direction was parallel to rolling direction).

The cutting process was conducted using a coolant. An end mill (211811) of GARANT (Munich, Germany) was used for research. Table 4 shows the specification of the milling cutter.

**Figure 2:**
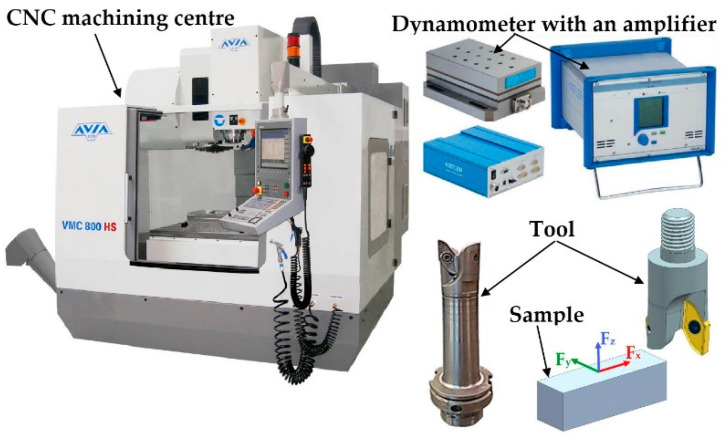
Schematic diagram of the methodology.

The measuring system used consisted of 9257B piezoelectric dynamometer of Kistler (Winterthur, Switzerland) connected to 5070A charge amplifier. The signal from the amplifier was sent to 5697A DAQ module and it was analysed using the dedicated DynoWare software (2825A, Kistler, Winterthur, Switzerland) [29]. Figure 2 presents the schematic diagram of the methodology.

Within the experiment, the analysis of the microstructures of the surface layer was also carried out for the tested relations between the milling direction and the rolling direction, as well as for the examined aluminium alloys. The Nikon Epiphot inverted metallographic microscope (Nikon, Tokyo, Japan) with the ToupView software was used. The aim of the analysis of the surface layer microstructures was to determine the thickness of the textured surface layer formed after rolling both EN AW-2017A T451 and EN AW-2024 T351. 

The tests were repeated five times. The number of repetitions was determined from Equation (1):

(1) $$n=\frac{t_{\left(\alpha ;f\right)}^{2}S_{x}^{2}}{\varepsilon^{2}}$$

where: $S_{x}^{2}$—variance of the tested variable determined on the basis of the preliminary test results ($S_{x}^{2}=$ 4.77), *ε*—assessment accuracy (*ε*—5% of obtained average value, *ε* = 2.5), *t*—critical value of the Student’s *t*-test (*t* = 2.262), *α*—significance level (*α* = 0.05), and *f*—number of degrees of freedom (*f* = 9).

## 3. Results

**Figure 3:**
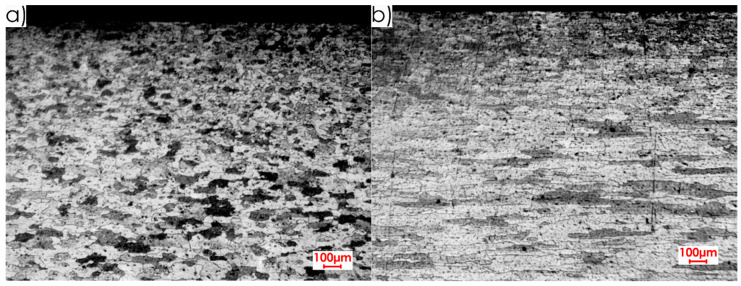
Microstructure of surface layer for EN AW-2017A T451 alloy: (**a**) perpendicular rolling direction, (**b**) parallel rolling direction (2.5× magnification).

**Figure 4:**
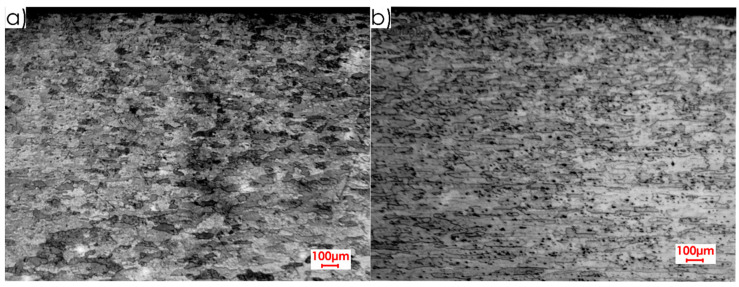
Microstructure of surface layer for EN AW-2024 T351 alloy: (**a**) perpendicular rolling direction, (**b**) parallel rolling direction (2.5× magnification).

The research began with the analysis of the microstructures of the surface layer formed after rolling for the tested relations between the milling direction and the rolling direction as well as for both aluminium alloys. Figure 3 and Figure 4 show the microstructure images for EN AW-2017A T451 and EN AW-2024, respectively.

Analysing microstructures (Figure 3 and Figure 4), a difference was found between the core and the surface layer. A clearly elongated grains were also observed in the rolling direction, especially in the core of the material. This statement is true for both alloys. In the case of EN AW-2017A T451, the textured surface layer is more visible (Figure 3) than for EN AW-2024 T351 (Figure 4). For EN AW-2017A T451, the grains in the area of the surface layer are significantly dense and fragmented in comparison to the material core and they do not have a clear orientation. A different situation can be observed in the case of the EN AW-2024 T351, i.e., there is no clear difference, especially in the perpendicular direction to the rolling direction, between the surface layer and the core. Some differences between the surface layer and the core for this alloy can be stated for the parallel direction to the rolling direction—the grains are finer and the structure is not clearly oriented in the surface layer. When comparing both alloys, it was found that the microstructure of EN AW-2017A T451 in the core is characterised by greater graininess and the thickness of surface layer is deeper (about 0.4 mm) in comparison to EN AW-2024 T351, where it is approximately 0.25 mm. 

Based on the microstructure analysis (Figure 3 and Figure 4), it was found that the thickness of the textured surface layer formed after rolling is from range 0.25–0.4 mm for EN AW-2017A T451 and EN AW-2024 T351 alloys. Hence, it was decided to remove the surface layer with the indicated values of depths of cut *a_p_* in the milling process. In the case of the cutting speed *v_c_*, the values were selected by trying to conduct “delicate machining” (at *v_c_* = 100 m/min) and at significantly higher values (*v_c_* = 1000 m/min and *v_c_* = 1500 m/min).

**Figure 5:**
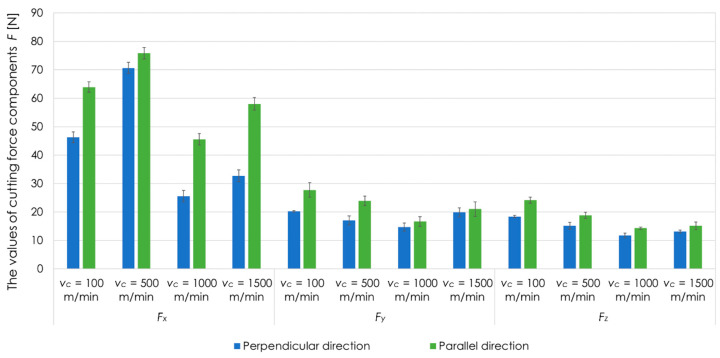
Cutting force components *F_x_*, *F_y_*, *F_z_*, for EN AW-2017A T451 aluminium alloy, tested cutting speeds *v_c_*, perpendicular and parallel milling direction in relation to the rolling direction, *a_p_* = 0.4 mm.

Figure 5 presents the values of the cutting force components *F_x_*, *F_y_*, *F_z_* for EN AW-2017A T451 alloy and analysed cutting speeds *v_c_* at *a_p_* = 0.4 mm. The recorded values of the cutting force components are the effect of various phenomena accompanying the cutting process. The study did not focus on all components of the cutting force and did not analyse the phenomena affecting their value, but only on determining the values of the cutting force depending on the selected technological parameters. In particular, it was bothered to establish for which cutting speed *v_c_*, the cutting force has the lowest value. Lower cutting force is the result of lower cutting resistance, and thus also lower deformations and post-machining residual stresses. In the further part of the paper, the analysis of the component of the cutting force *F_x_*, which had the highest values, in comparison to *F_y_* and *F_z_*, was made.

**Figure 6:**
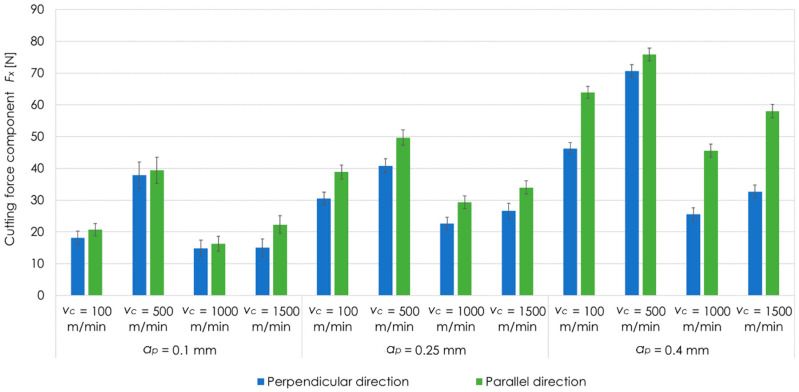
Cutting force component *F_x_* for EN AW-2017A T451 aluminium alloy, tested cutting speeds *v_c_*, depths of cut *a_p_* as well as perpendicular and parallel milling direction in relation to the rolling direction.

Figure 6 presents the results of cutting force component *F_x_* for EN AW-2017A T451 aluminium alloy, tested cutting speeds *v_c_*, depths of cut *a_p_* as well as perpendicular and parallel milling direction in relation to the rolling direction. Analysing the results, it was found that the highest value of cutting force component *F_x_* was recorded at *v_c_* = 500 m/min, while the lowest one at *v_c_* = 1000 m/min. These dependencies are true for all tested depths of cut *a_p_*. For perpendicular rolling direction and *a_p_* = 0.1 mm, value of cutting force component *F_x_* at *v_c_* = 1000 m/min was lower by over 60% than at *v_c_* = 500 m/min. For *a_p_* = 0.25 mm, the analogous difference was 40%, while for *a_p_* = 0.4 mm, it was about 60%. For parallel rolling direction and *a_p_* = 0.1 mm, the cutting force component *F_x_* was lower by about 60% at *v_c_* = 1000 m/min compared to *v_c_* = 500 m/min. In the case of *a_p_* = 0.25 mm and *a_p_* = 0.4 mm, the differences were about 40%. Additionally, it was observed that higher values of the cutting force component *Fx* were obtained for parallel milling direction in comparison to perpendicular direction.

**Figure 7:**
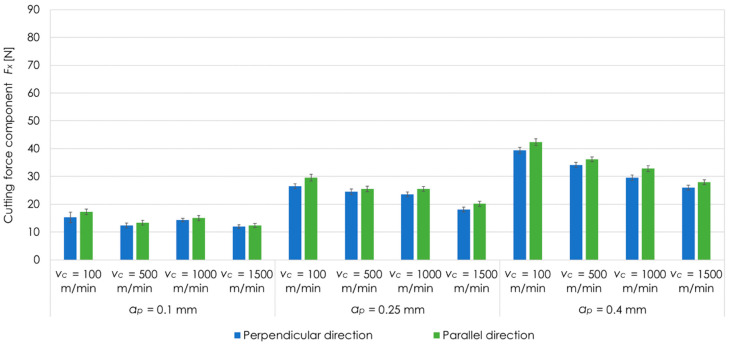
Cutting force component *F_x_* for EN AW-2024 T351 aluminium alloy, tested cutting speeds *v_c_*, depths of cut *a_p_* as well as perpendicular and parallel milling direction in relation to the rolling direction.

Figure 7 presents the results of cutting force component *F_x_* for EN AW-2024 T351 aluminium alloy, analysed values of cutting speeds *v_c_*, depths of cut *a_p_* as well as perpendicular and parallel milling direction in relation to the rolling direction. On the basis of the results, it can be seen that for EN AW-2024 T351, the maximum values of the cutting force component *F_x_* were noted at *v_c_* = 100 m/min, and the minimum one at *v_c_* = 1500 m/min for all tested depths of cut *a_p_*. For perpendicular rolling direction and *a_p_* = 0.1 mm, value of cutting force component *F_x_* at *v_c_* = 100 m/min was higher by almost 30% in comparison to *v_c_* = 1500 m/min. For *a_p_* = 0.25 mm, it was 45%, while for *a_p_* = 0.4 mm, it was over 50%. The analogous dependence was reached for parallel rolling direction. For *a_p_* = 0.1 mm, the cutting force component *F_x_* was higher by almost 40% at *v_c_* = 100 m/min compared to *v_c_* =1500 m/min. In the case of *a_p_* = 0.25 mm and *a_p_* = 0.4 mm, the differences were about 45% and 50%, respectively. It was also observed that higher values of the cutting force component *F_x_* were received for parallel milling direction than for perpendicular direction.

**Figure 8:**
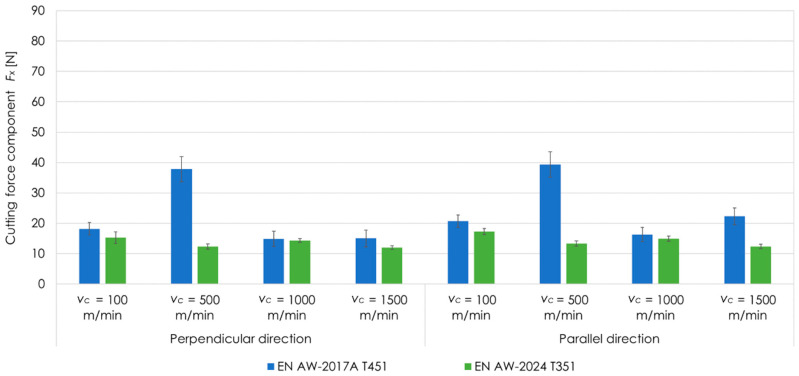
Comparison of cutting force component *F_x_* as a function of cutting speed *v_c_* for tested aluminium alloys as well as perpendicular and parallel milling direction in relation to the rolling direction at *a_p_* = 0.1 mm.

Figure 8 shows the comparison of cutting force component *F_x_* as a function of cutting speed *v_c_* for tested aluminium alloys (EN AW-2017A T451 and EN AW-2024 T351) as well as perpendicular and parallel relations between cutting tool feed direction and rolling direction at *a_p_* = 0.1 mm. On the basis of the results presented, it can be seen that for EN AW-2024 T351, the values of the cutting force component *F_x_* were lower in comparison to EN AW-2017A T451 for all examined cutting speeds *v_c_* and both relations between milling direction and rolling direction. 

On the basis of changes in the cutting force as a function of the cutting speed *v_c_* (Figure 5, Figure 6, Figure 7 and Figure 8), it is possible to determine the limit of occurrence of High Speed Cutting. The original definition of HSC stated that it is machining at cutting speeds *v_c_* 5–10 times, depending on the material to be machined, higher than the values used for conventional machining. Now there are new proposals for defining HSC. One of them introduces the concept of the so-called limit cutting speed *v_cgr_*, from which the HSC range begins. Different criteria for the moment of HSC occurrence are applied. Some researchers assume that the HSC can be discussed when an increase in the cutting speed *v_c_* causes a decrease in the cutting forces, in the case of the research conducted, this limit corresponds to the speed *v_c_* = 500 m/min for EN AW-2017A T451 and *v_c_* = 100 m/min for EN AW-2024 T351. In other studies, it is assumed that the limit speed is the cutting speed to which the cutting force decreases significantly and then increases gently. In the case considered, this is speed of about *v_c_* = 1000 m/min for EN AW-2017A T451 and *v_c_* = 1500 m/min for EN AW-2024 T351. The second way of defining HSC, especially when machining thin-walled parts, appears to be more appropriate, as it is important to minimise the cutting forces when machining this type of parts. It can therefore be determined that when machining such elements one ought to use HSC, ensuring minimisation of cutting forces.

An inseparable element of measurement is its uncertainty. It characterises the dispersion of the results obtained and it can be determined on the basis of two methods, i.e., A and B. The uncertainty component of A method is based on the statistical analysis of the measurement results, i.e., the calculation of the standard deviation of the mean, while the uncertainty component of B method takes into account the data included in the calibration certification of the measuring instrument used [3,30].

The estimation procedure of expanded uncertainty *U* consists in to multiply the combined standard uncertainty *u_c_* by the expansion factor, in this case *k* = 2, according to Equation (2).

(2) $$U=\pm ku_{c}$$

Calculating the combined standard uncertainty *u_c_*, it is necessary to take into account the component of standard uncertainty determined by A method *u_A_* and the components of the standard uncertainty assessed by B method *u_Bi_* (Equation (3)).

(3) $$u_{c}=\pm \sqrt{u_{A}^{2}+\overset{n}{\underset{i=1}{\sum }}u_{Bi}^{2}}$$

In the case of measurement uncertainty component estimated by using B method, two factors should be taken into account, that is, resulting from the linearity *u_Bl_* as well as from the hysteresis *u_Bh_* (Equation (4)).

(4) $$\overset{n}{\underset{i=1}{\sum }}u_{Bi}^{2}=u_{Bl}^{2}+u_{Bh}^{2}$$

The combined standard uncertainty *u_c_* is therefore estimated according to Equation (5), having regard to all uncertainty components both from A and B methods.

(5) $$u_{c}=\pm \sqrt{u_{A}^{2}+u_{Bl}^{2}+u_{Bh}^{2}}$$

As already mentioned, the uncertainty component estimation by B method is based on the data given in the calibration certificate of the instrument used, in this case the piezoelectric dynamometer. The standard uncertainty components resulting from linearity *u_Bl_* is determined from Equation (6) and from the hysteresis *u_Bh_* from Equation (7). In both cases, a rectangular distribution of measurement results was assumed.

(6) $$u_{Bl}=\pm \frac{1\%\cdot FSO}{\sqrt{3}}$$

(7) $$u_{Bh}=\pm \frac{0.5\%\cdot FSO}{\sqrt{3}}$$

Table 5 and Table 6 present estimated uncertainties and their components of A and B methods for perpendicular and parallel milling direction in relation to the rolling direction, selected depth of cut *a_p_* = 0.4 mm as well as tested EN AW-2017A T451 and EN AW-2024 T351 aluminium alloys, respectively. 

In other cases, the uncertainty was determined analogously. Summarising, for EN AW-2017A T451 the values of expanded uncertainty *U* ranges from 7 to 11 N, while for EN AW-2024 T351—around 6.5 N. The uncertainty components from B method are constant for all variables. They result from the data available in the calibration certificate. The differences appear in the case of A method, which takes into account the standard deviation of the mean.

## 4. Conclusions

The conducted research and analysis of results allow to formulate the following conclusions:

- The textured surface layer after rolling was removed during milling. This type of pre-machining is often performed in industrial conditions and it is aimed at preventing of deformations of the manufactured elements, especially thin-walled ones. It is important to generate the lowest possible post-machining residual stresses. The values of these stresses are correlated, among others, with cutting resistance and therefore cutting force. The lowest cutting force values were obtained for the EN AW-2017A T451 alloy at *v_c_* = 1000 m/min, and for the EN AW-2024 T351 alloy at *v_c_* = 1500 m/min, respectively.
- On the basis of results, it can also be stated that for each of applied depth of cut *a_p_*, the lowest values of cutting forces as a function of cutting speed *v_c_* for individual alloys were always obtained for the same cutting speed *v_c_*. It can be concluded that the minimum value of the cutting force as a function of cutting speed *v_c_* is a characteristic value for a given material.
- The cutting speed *v_c_* for which the minimum cutting force value is obtained is a limit parameter that defines the transition from conventional machining to the High Speed Cutting. Therefore, it can be assumed that for EN AW-2017A T451 alloy, HSC occurs at *v_c_* = 1000 m/min, and for EN AW-2024 T351 at *v_c_* = 1500 m/min.
- It was also found that during milling with parallel feed direction of the cutting tool to rolling direction, the generated cutting forces were higher than for the perpendicular direction. The clear difference in the values of these forces results from the fact that only the surface layer, which is characterised by a significant anisotropy of properties in these two directions, was cut. It is also seen in the microstructure images.
- According to literature data and previously research results obtained, materials characterised by lower strength, stiffness and greater plasticity have a higher cutting speed *v_c_* limit at which HSC occurs. From these two aluminium alloys tested, the EN AW-2017A T451 alloy is undoubtedly such a material. However, this alloy is characterised by a lower cutting speed *v_c_* limit. Therefore, it should be considered whether it may be caused by greater stresses and deformations of the surface layer compared to the EN AW-2024 T351, which translated into hardening and strengthening of the surface layer of the EN AW-2017A T451. This thesis may be confirmed by higher cutting forces for the EN AW-2017A T451 in comparison to EN AW-2024 T351.

## Figures and Tables

**Table 1:** The chemical composition and selected properties of the EN AW-2017A T451 alloy [26,27].

**Chemical Composition (%)**									
Si	Fe	Mg	Cu	Mn	Cr	Zn	Zr + Ti	Other	Al
0.2–0.8	≤0.7	0.4–1.0	3.5–4.5	0.4–1.0	≤0.1	≤0.25	≤0.25	≤0.15	Rest
**Selected Properties**									
Density *ρ* (g/cm^3^)		Young modulus *E* (GPa)		Tensile strength *R_m_* (MPa)		Yield strength *R_p0.2_* (MPa)		Brinell hardness (HB)	
2.79		72.5		390		250		110	

**Table 2:** The chemical composition and selected properties of the EN AW-2024 T351 alloy [26,27].

**Chemical Composition (%)**										
Si	Fe	Mg	Cu	Mn	Cr	Zn	Zr + Ti	Ti	Other	Al
≤0.5	≤0.5	1.2–1.8	3.8–4.9	0.3–0.9	≤0.1	≤0.25	≤0.2	≤0.15	≤0.15	Rest
**Selected Properties**										
Density *ρ* (g/cm^3^)		Young modulus *E* (GPa)		Tensile strength *R_m_* (MPa)		Yield strength *R_p0.2_* (MPa)		Brinell hardness (HB)		
2.78		73		469		324		120		

**Table 3:** The technological parameters used.

Technological Parameters			
Variable Depth of Cut *a_p_* (mm)	Milling Width *a_e_* (mm)	Feed Per Tooth *f_z_* (mm/tooth)	Variable Cutting Speed *v_c_* (m/min)
-	20	0.05	100
0.1			500
0.25			1000
0.4			1500

**Table 4:** Specification of milling cutter [28].

Symbol	GARANT 211811
External diameter *D* (mm)	32
Number of teeth *z*	2
Overall length *L* (mm)	47
Helix angle (°)	8
Rake angle (°)	25
Flank angle (°)	7
Cutting insert	VCGX 220508 FR HU 7810 (211856)

**Table 5:** Uncertainty and its components for EN AW-2017A T451, perpendicular and parallel milling direction in relation to the rolling direction as well as *a_p_* = 0.4 mm.

Uncertainty	*v_c_* = 100 m/min		*v_c_* = 500 m/min		*v_c_* = 1000 m/min		*v_c_* = 1500 m/min	
	⊥	∥	⊥	∥	⊥	∥	⊥	∥
*u_A_* (N)	2.07	1.99	4.15	4.14	2.56	2.37	2.76	2.77
*u_Bl_* (N)	2.88	2.88	2.88	2.88	2.88	2.88	2.88	2.88
*u_Bh_* (N)	1.44	1.44	1.44	1.44	1.44	1.44	1.44	1.44
*u_c_* (N)	3.83	3.79	5.25	5.24	4.11	4.00	4.24	4.25
*U* (N)	7.66	7.57	10.51	10.49	8.23	8.00	8.48	8.49

Note: ⊥—milling direction was perpendicular to the rolling direction; ∥—milling direction was parallel to the rolling direction.

**Table 6:** Uncertainty and its components for EN AW-2024 T351, perpendicular and parallel milling direction in relation to the rolling direction as well as *a_p_* = 0.4 mm.

Uncertainty	*v_c_* = 100 m/min		*v_c_* = 500 m/min		*v_c_* = 1000 m/min		*v_c_* = 1500 m/min	
	⊥	∥	⊥	∥	⊥	∥	⊥	∥
*u_A_* (N)	1.17	1.26	0.90	0.87	0.95	0.99	0.89	0.87
*u_Bl_* (N)	2.88	2.88	2.88	2.88	2.88	2.88	2.88	2.88
*u_Bh_* (N)	1.44	1.44	1.44	1.44	1.44	1.44	1.44	1.44
*u_c_* (N)	3.43	3.46	3.34	3.34	3.36	3.37	3.34	3.34
*U* (N)	6.85	6.92	6.69	6.67	6.71	6.74	6.68	6.67

Note: ⊥—milling direction was perpendicular to the rolling direction; ∥—milling direction was parallel to the rolling direction.
